# Systematic review of amino acid profiles among COVID-19 patients caused by SARS-CoV-2

**DOI:** 10.1007/s40200-026-02012-4

**Published:** 2026-07-16

**Authors:** Saber Soltani, Hamid Choobineh, Fariba Nabatchian, Mohammad Kord, Bahram Nikmanesh, Farideh Razi, Houran Firouzian, Ziba Majidi

**Affiliations:** 1 Department of Medical Laboratory Science, School of Allied Medical Sciences, Tehran University of Medical Sciences, Tehran, Iran; 2 Zoonosis Research Center, Tehran University of Medical Sciences, Tehran, Iran; 3 Metabolomics and Genomics Research Center, Endocrinology and Metabolism Molecular-Cellular Sciences Institute, Tehran University of Medical Sciences, Tehran, Iran

**Keywords:** Amino acid dysregulation, SARS-CoV-2, COVID-19 pathogenesis, Metabolic biomarkers, Branched-chain amino acids (BCAAs), Urea cycle, Metabolomics

## Abstract

**Background:**

Emerging evidence highlights the critical role of amino acid metabolism in the pathogenesis and severity of COVID-19. This review included studies encompassing patients with varying degrees of disease severity, from mild to critical cases.

**Methods:**

A systematic review following PRISMA guidelines was conducted, and study quality was appraised using the Newcastle–Ottawa tools to explore the relationship between amino acid profiles and clinical outcomes in COVID-19 patients. A comprehensive search of PubMed, Scopus, Web of Science, and Google Scholar (from the start of the pandemic through the most recent data available on December 2024) identified peer-reviewed studies reporting original data on amino acid metabolism in COVID-19 patients.

**Results:**

Fourteen eligiblestudies involving 1355 confirmed COVID-19 patients (1726 total participants) were included and revealed significant disruptions inamino acid profiles. Key findings included reduced levels of arginine, glutamine, and tryptophan, alongside elevated phenylalanine andbranched-chain amino acids (BCAAs). These changes were associated with disease severity, immune suppression, systemicinflammation, and metabolic reprogramming. Dysregulation of pathways such as the urea cycle and kynurenine pathway were linked toendothelial dysfunction and immune dysregulation.

**Conclusions:**

Dysregulated amino acid profiles may serve as potential biomarkers for disease severity and require further validation before clinical application. Restoring amino acid balance or modulating metabolic pathways could improve clinical outcomes. Further research is needed to validate these findings and explore personalized treatment strategies aimed at mitigating the metabolic consequences of SARS-CoV-2 infection.

**Supplementary Information:**

The online version contains supplementary material available at 10.1007/s40200-026-02012-4.

## Introduction

The Severe Acute Respiratory Syndrome Coronavirus 2 (SARS‑CoV‑2) was responsible for the initial outbreak of coronavirus disease 2019 (COVID-19) [1]. Due to its significant morbidity and mortality, the pandemic became a global issue. Therefore, the interactions between host and pathogen contribute to understanding metabolic dysregulation that affects viral pathogenesis [2].

Amino acids, as structural components of proteins, play crucial roles in maintaining physiological homeostasis and metabolic changes [3]. Besides their primary role in protein synthesis, amino acids may participate in the regulation of the immune system, redox balance, neurotransmitter production, and signaling pathways, as a key mediator [4, 5]. Viral infections such as COVID-19 may cause further disruption in metabolic pathways, resulting in systemic inflammation, endothelial damage, and metabolic reprogramming that leads to severe outcomes [6, 7]. A combination of factors is involved in alterations of amino acid metabolism, including direct viral interference with host cell machinery, immune-mediated catabolism, and the systemic stress response [8–10].

Prior evidence suggests that SARS-CoV-2 utilizes host amino acid metabolism pathways which are critical for the immune system function and tissue repair, to facilitate viral replication [11, 12]. Studies have demonstrated the association between arginine downregulation and impaired NO production, and its potential role in endothelium dysfunction and vascular complications which is observed in severe COVID-19 patients [13]. Similarly, elevation in tryptophan metabolism via the kynurenine pathway in severe cases, may lead to the accumulation of neuroactive metabolites and correlates with immune suppression and subsequent neurological manifestations [14, 15]. Dysregulation of leucine, isoleucine, and valine levels, which are also known BCAAs, has been reported as either elevated or reduced in blood circulation, reflecting muscle catabolism or hepatic dysfunction [16]. Also, accumulating evidence suggests that persistent metabolic and lipidomic disturbances in patients with Post-COVID-19 Condition may contribute to the prolonged course of their symptoms [17]. The identified metabolic signature may serve as a promising biomarker for clinical decision-making and a potential target for Long COVID-19 treatment [18].

Amino acid profile alterations may serve as a potential biomarker of disease severity and provide diagnostic insights into the complex association between viral pathogenesis and host metabolism. Understanding these metabolic disruptions may provide novel therapeutic targets and strategies for earlier intervention.

Although several studies have investigated the role of metabolomics, a comprehensive systematic review which summarizes amino acid alterations associated with COVID-19 and their links to disease severity, immune function, and metabolic regulation, has not been carried out. Therefore, this study specifically aims to (1) systematically review current evidence on amino acid dysregulation in COVID-19 (2), identify potential metabolic biomarkers associated with disease severity, and (3) explore therapeutic implications of amino acid metabolism in infection outcomes.

### Methodology

This systematic review was conducted based on the PRISMA guidelines and aimed to evaluate the association between amino acid profiles and metabolic alterations in COVID-19 patients. The PRISMA flowchart discusses the specific exclusion criteria at the full-text screening stage including unrelated outcomes, insufficient data, or animal studies. Due to the retrospective nature, this review was not registered in PROSPERO; however, inclusion and exclusion criteria were defined before the data extraction.

#### **Eligibility Criteria**

Studies which investigated metabolic changes, specifically amino acid profiles or metabolism, in patients diagnosed with COVID-19, reported original data on amino acid metabolism or the association with clinical outcomes, and were peer-reviewed articles were included in this review.

Studies that were not directly related to COVID-19 or SARS-CoV-2 infection, articles that did not provide primary data, and studies conducted on animal models or in vitro experiments were excluded.

#### **Information Sources**

A comprehensive literature search was performed across multiple databases, including PubMed, Scopus, Web of Science, and Google Scholar. The final literature search was conducted on 31 December 2024.

Grey literature, including preprints and conference abstracts, was screened as additional source, for plausible relevance but only peer-reviewed studies were included in the final analysis. Also, to identify the eligibility of studies, reference lists of relevant articles, and manual searches of key journals, were reviewed. No additional studies meeting the inclusion criteria were identified beyond those retrieved through the database search.

The search was limited to articles published in English from the outbreak of the SARS-CoV-2 through the most recent data available in December 2024.

#### **Search Strategy**

The search strategy consists of both keyword and MeSH terms. The following search terms were used:

(COVID-19 OR “corona virus” OR “2019-nCoV Infection” OR “COVID-19 Pandemic” OR “sars covid2” OR “SARS-CoV-2 Infection” OR “SARS-CoV-2”) AND (“profile of amino acid” OR “amino acid” OR “amino acid profile” OR “amino acid metabolism”) AND (Metabolism OR “Metabolic Concept” OR “metabolic changes” OR “metabolomics”).

#### **Study Selection Process**

**Fig. 1:**
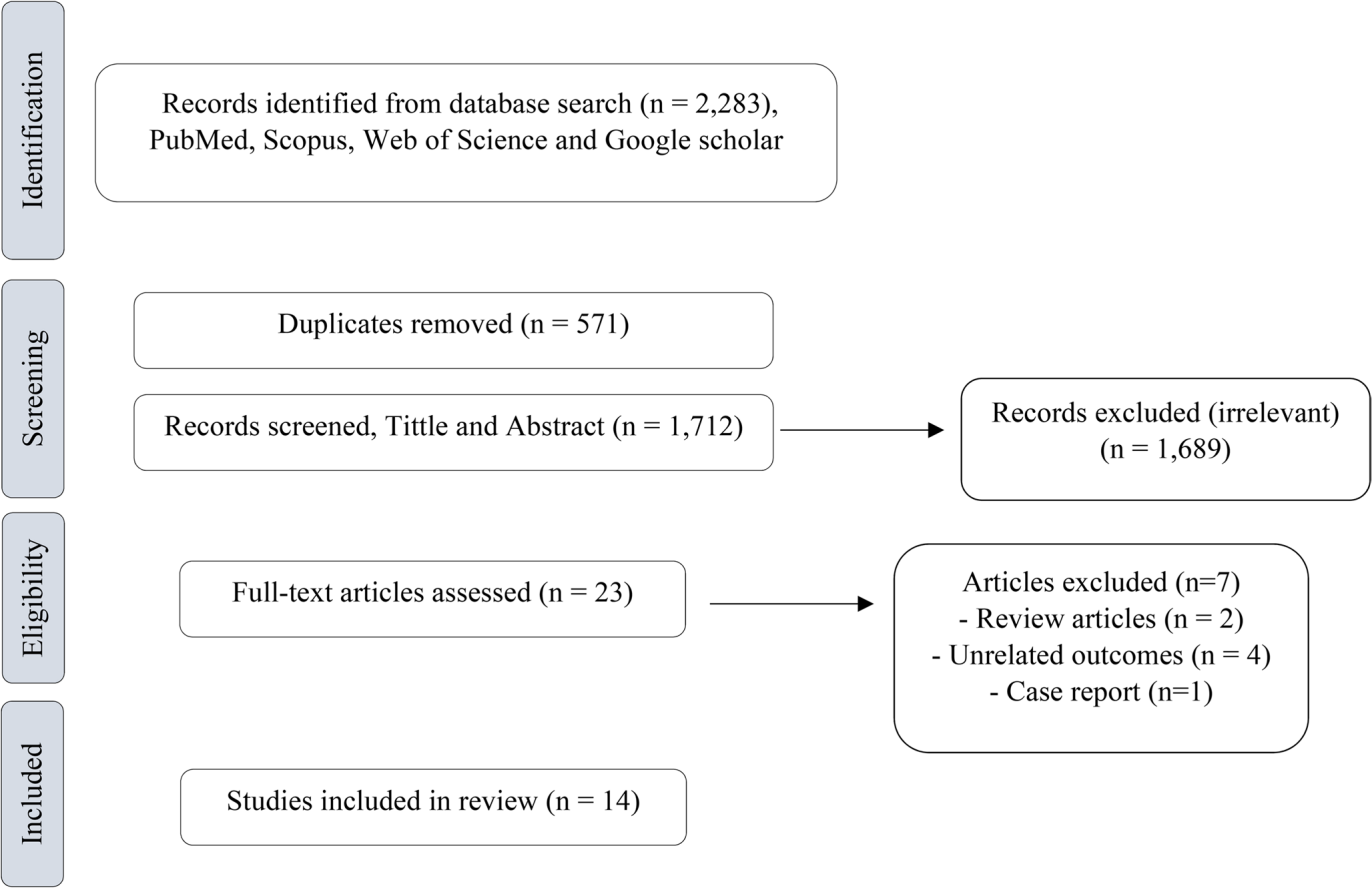
Study selection flow chart for studies in the systematic review

After performing the initial search, a total of 2,283 articles were identified. The 571 duplicate articles were removed. A two-step screening process was conducted on the remaining 1712 articles. First, titles and abstracts were reviewed to exclude irrelevant articles. Secondly, the eligible articles based on their title and abstract were progressed to the full-text screening. Eventually, 14 articles met the inclusion criteria and were included in this systematic review as it illustrated in Fig. 1. Any disagreements during the selection process were resolved by consultation among the review team.

#### **Data Extract**

A standardized form was designed to systematically extract data and key information from each included study. Key characteristics included author, year of publication, study design, participant demographics, details on amino acid profiles, observed metabolic changes, and clinical outcomes related to COVID-19. Data extraction was performed independently by two reviewers, and any conflicts were settled through discussion, and for the final decision, a third reviewer was consulted or the decision of a third reviewer.

#### **Quality Assessment**

All 14 included studies were observational in design (cohort, case-control, or cross-sectional); therefore, the Newcastle-Ottawa Scale (NOS) was used to assess the methodological quality of every included study. Each study was independently scored by two reviewers based on the appropriate criteria, including selection, comparability, and outcome/exposure assessment, and any differences were resolved through discussion. The detailed quality assessment results are described in Table 1, which presents the NOS domain scores and overall quality ratings for each included study. Although most included studies did not report their funding or potential conflicts of interest, this may have affected the risk of bias, which should be considered when interpreting the results.

**Table 1:** Quality assessment of included studies using Newcastle–Ottawa scale and cochrane risk of bias tool

Study (Author, Year)	Study type	Assessment tool	Selection	Comparability	Outcome/Exposure	Total score / Risk	Overall quality
Alptug Atila (2021)	Case–control	NOS	3	0	2	5/9	Low
Chris A. Rees (2021)	Observational	NOS	3	0	3	5/9	Low
Eva Baranovicova (2021)	Longitudinal	NOS	3	2	2	7/9	Moderate
Gagandeep Kaur (2021)	Comparative	NOS	2	1	2	5/9	Low
Lomova NA (2021)	Observational	NOS	3	2	2	7/9	Moderate
Zili Zhang (2021)	Observational	NOS	2	2	2	6/9	Moderate
Ali Ozturk (2022)	Case-control	NOS	3	0	3	6/9	Moderate
Anthony T. Le (2022)	Case-control	NOS	2	2	2	6/9	Moderate
Lomova NA (2022)	Observational Case-Control	NOS	3	2	2	7/9	Moderate
Merve Ergin Tuncay (2022)	Case–control	NOS	3	0	2	5/9	Low
Sedat Özbay (2023)	Cross-sectional	NOS	3	2	2	7/9	Moderate
Hüseyin Aydın (2023)	Observational	NOS	3	2	2	7/9	Moderate
Ina Maltais-Payette (2023)	Observational	NOS	4	2	3	9/9	High
Siqi Ming (2023)	Case-control (cross-sectional)	Cochrane	2	0	2	4/9	Low

#### **Data Synthesis**

To sum up the key findings about amino acid metabolism and its association with COVID-19 manifestations, a qualitative synthesis of the data was performed.

A narrative synthesis approach was used to address the expected heterogeneity among the included studies and ensure a balanced interpretation of the findings. Due to heterogeneity in study designs, populations, and methodologies, a meta-analysis was not likely to be achieved. Instead, results were synthesized narratively, highlighting patterns, discrepancies, and gaps in the existing evidence.

#### **Reporting Standards**

This systematic review was conducted according to the PRISMA guidelines for reporting systematic reviews.

## Results

### Study characteristics and methodological overview

A total of 14 studies with 1355 confirmed COVID-19 patients (total 1726 participants ; 915 males and 811 females ) met the inclusion criteria for this systematic review [12, 19–31] The characteristics of the included studies such as study design, sample size, age and sex are detailed in Table 2. The clinical manifestations of included patients, extended a broad spectrum from asymptomatic to sever COVID-19 cases. Significant association between disease severity and clinical outcomes, was demonstrated in all studies. Clinical samples comprised serum samples (4 studies), plasma sample (9 studies), and respiratory swabs (1 study). Metabolic profile of patients was evaluated by liquid chromatography - tandem mass spectrometry (LC–MS/MS) in 13 studies and the remaining study used Nuclear Magnetic Resonance (NMR) [30].

**Table 2:** Characteristics of included studies

Author(s)	Study design	Sample size	Age (mean ± SD, median or range)	Sex distribution	Confounding factors considered	Sample type	Sampling time
Alptug Atila (2021)	Case-control study	111 patients; 30 controls	18–85	85 Males/ 56 Females	Age, sex, comorbidities	Serum	At admission
Chris A. Rees (2021)	Prospective observational study	52 patients; 28 controls	3–99	39 Male/ 41 Female	Comorbidities reported	Plasma	During hospitalization for COVID-19 or MIS-C
Eva Baranovicova (2021)	Longitudinal metabolomic study	53 patients; 55 controls	> 65	60 Male / 48 Female	Dexamethasone treatment, patient diet (diabetic vs. non-diabetic)	Plasma	Days 1, 3, and 7 after hospital admission
Gagandeep Kaur (2021)	Comparative metabolomic study	6 COVID-19-positive patients, 6 COVID-19-recovered subjects	COVID-19-positive: 47; COVID-19-recovered: 36.33	7 Male/ 5 Female	Smoking status, but no significant differences were observed	Serum	Not specified (samples collected between April–May 2020)
Lomova NA (2021)	Observational Case-control study	29 pregnant women with COVID-19, 17 healthy controls	> 29.9	0 Male/ 46 female	Multiple pregnancies, Rh or ABO isoinmunization, chromosomal abnormalities, genetic mutations, and congenital malformations were excluded.	Amniotic fluid and cord blood plasma	During delivery
Zili Zhang, (2021)	Observational	5 patients; 5 controls	63–75	7 males/ 3 females	Age, sex, comorbidities	Airway mucus	During hospitalization (January 26, 2020, to February 15, 2020)
Ali Ozturk (2022)	Case-control	38 patients; 30 controls	30 − 95	30 Male /38 Female	Age, sex, comorbidities	Serum	At admission
Anthony T. Le.(2022)	Case-control + machine learning diagnostic model	70 patients, 130 control	32.5–67	95 Male / 82 Female	Age, Sex, CRP, PCT	Plasma	Within 7 days of infection
Lomova NA (2022)	Observational Case-Control study	29 pregnant women with COVID-19, 17 healthy controls	Controls: 32 COVID-19: 29.9	0 Male/ 46 female	Exclusion criteria included multifetal pregnancy, rhesus- and ABO-isolmmunizations, chromosome aberrations, genetic mutations, and congenital malformations in the fetus.	Maternal venous blood plasma, amniotic fluid, umbilical cord blood plasma	During pregnancy and at delivery
Merve Ergin Tuncay (2022)	case control	116 patients; 46 controls	COVID-19 patients: 60.8 ± 10.8, Control Group: 37.5 ± 10.1	86 Male/ 76 Female	Age, sex	serum	At admission
Sedat Özbay et al. (2023)	Cross-Sectional	35 patients; 35 controls	patient: 48.5 ± 14.9, control groups: 48.8 ± 14.6 years	45 Male / 25 Female	Age, sex	Plasma	At admission
Hu¨seyin Aydın (2023)	Observational Case-Control	35 patients; 35 controls	19–74	45 Male / 25 Female	Age, Sex, Comorbidities	Plasma	At time of diagnosis
Ina Maltais-Payette (2023)	Observational	736 patients	44.2–81.9	416 Male/ 320 Female	Age, Sex, BMI, Comorbidities, Medication	Plasma	Within 7 days of inclusion
Siqi Ming (2023)	Observational and experimental (in vitro and in vivo)	40 patients; 10 controls	Not specified	Not specified	-	Plasma, mouse tissues	Admission, progressive, and remission stages of COVID-19

### Amino acid dysregulation in COVID-19

Dysregulated amino acid profile was documented in all 14 studies. Downregulated levels of Arginine, Glutamine and Tryptophan among severe COVID-19 patients were reported in several investigations. This pattern reflects impaired immune and endothelial function. In contrast upregulated pattern of phenylalanine and BCAAs suggests enhanced protein catabolism and systemic inflammation. Despite this overall pattern, in limited number of studies [25, 29] divergent results were reported. These discrepancies, attributed to variations in disease stage, comorbidities, or methodological differences.

#### Arginine and urea cycle metabolism

The metabolomic data of included studies have revealed a dysregulation in arginine metabolism pathway. This pathway disruption is associated with endothelial dysfunction, impaired nitric oxide synthesis, and T-cell dysregulation. A significant reduction in arginine levels along with ornithine, citrulline, glutamine, alanine, histidine, proline, serine, tryptophan, and tyrosine, was recorded in COVID-19 patients by Rees et al., alongside a marked elevation in Phenylalanine levels [12]. Sedat Özbay et al.focused on dysregulations in Urea cycle and documented reduced levels of arginine, arginosuccinate, aspartic acid, citrulline, glutamine, lysine, and ornithine [22]. Le et al. reported reduced arginine levels [24] accompanied by increased aspartic acid and sulfocysteine. Further evidence provided by Lomova NA from multiple samples comprising amniotic fluid, maternal plasma, and umbilical cord plasma supports the hypothesis that the arginine metabolism pathway is impaired in COVID-19 [28]. Amino acid alterations, related pathways, and their association with clinical outcomes are summarized in Table 3.

**Table 3:** Summary of amino acid alterations and pathway involvement

Author(s)	Measurement method	Pathways involved	Altered amino acids	Correlation with clinical outcomes	Key findings
Alptug Atila (2021)	LC-MS/MS	Immune response, metabolic pathways, amino acid catabolism	L-2-aminobutyric acid, L-phenylalanine(↑). Taurine, trans-4-hydroxy L-proline, L-proline, L-threonine, L-glutamine, L-histidine, L-citrulline(↓).	Yes	L-2-aminobutyric acid and L-phenylalanine were identified as potential biomarkers for COVID-19 diagnosis and prognosis. BCAAs (leucine, isoleucine, valine) showed a high correlation with each other and decreased in severe patients. Decreased levels of taurine, L-glutamine, and L-threonine may indicate immune dysfunction and metabolic dysregulation in COVID-19 patients.
Chris A. Rees (2021)	LC-MS/MS	Arginine metabolism, endothelial dysfunction, nitric oxide synthesis	Arginine, Ornithine, Citrulline, Glutamine, Alanine, Histidine, Proline, Serine, Tryptophan, Tyrosine (↓). Phenylalanine (↑).	Yes	Low arginine bioavailability was observed in both adults and children with COVID-19/MIS-C when compared to controls. Increased arginase activity was suggested by the low arginine-to-ornithine ratio and global arginine bioavailability ratio (GABR).
Eva Baranovicova (2021)	NMR spectroscopy	Energy metabolism, TCA cycle, protein catabolism, immune response	BCAAs, phenylalanine, Phe/Tyr ratio (↑) glutamine (↓)	Yes	↑ Glucose and 3-hydroxybutyrate levels were observed, indicating increased production of ketone bodies as an alternative energy source. ↓ Citrate levels were decreased, suggesting alterations in the TCA cycle. ↑ BCAAs levels increased, representing accelerated protein catabolism and offering an additional energy source. ↓ Glutamine levels decreased initially but normalized faster in survivors compared to non-survivors. ↑ The Phe/Tyr ratio was elevated and more pronounced in non-survivors, reflecting exacerbated and generalized inflammatory processes.
Gagandeep Kaur (2021)	Ultra-performance liquid chromatography/tandem mass spectrometry (UPLC-MS)	Tryptophan metabolism, lipid metabolism, amino acid metabolism	tryptophan, indole, indole-3-acrylic acid, glycine, leucine, guanidine acetic acid, L-2-amino-3-oxobutanoic acid, (S)-methylmalonate-semialdehyde (↑) NS-ethyl-L-glutamine, urocanic acid (↓)	-	Increased levels of phospholipids, including sphingomyelins, phosphatidylcholines, and arachidonic acid, were observed in COVID-19-positive patients compared to recovered subjects. Additionally, tryptophan and its metabolites, such as indole and indole-3-acrylic acid, showed increased levels in the serum of COVID-19-positive patients.
Lomova NA (2021)	HPLC-MS	Amino acid metabolism, protein production programming	Beta-alanine (↑), methylhistidine, Arginine, Cystathionine, Cystine, Glutamine, Histidine and Trans-4-hydroxyproline Cord Blood Plasma: 1-methylhistidine, Cystine, Histidine (↓)	Yes	Significant differences in amino acid concentrations were observed in the amniotic fluid and cord blood plasma of COVID-19 patients when compared to healthy controls. These alterations suggest potential impairments in fetal metabolome and protein production programming.
Zili Zhang, (2021)	Proteomic analysis (LC-MS/MS), GO and KEGG pathway analysis	Amino acid degradation, amino acid metabolism, oxidative phosphorylation, phagosome, cholesterol metabolism, complement and coagulation cascades, lysosome	Valine, leucine, isoleucine, beta-alanine, tryptophan, cysteine, methionine, phenylalanine, arginine, tyrosine	Elevated D-dimer, white blood cell count, IL-6, and lactate dehydrogenase levels correlated with severe COVID-19.	92 Differentially Expressed Proteins (DEPs): Identified in COVID-19 patients, these proteins are enriched in metabolic and complement/coagulation pathways. 375 Unique Proteins: Specifically present in COVID-19 patients.
Ali Ozturk (2022)	LC-MS/MS	Metabolic regulation, immune response	Alanine, Arginine, Glutamine, Asparagine, Aspartic acid, Citrulline, Glutamic acid, Glycine, Histidine, Leucine, Isoleucine, Alloisoleucine, Lysine, Methionine, Ornithine, Phenylalanine, Proline, Valine, Alpha-aminoadipic acid, Anserine, Beta-aminoisobutyric acid, Beta-alanine, 1-Methylhistidine, 3-Methylhistidine, Serotonin, Ethanolamine, Taurine (↑),Hydroxyproline, Sarcosine, Alpha-aminopimelic acid(↓) .-	Yes	Increase in BCAAs such as leucine, isoleucine, and valine, potentially linked to muscle catabolism in patients with myalgia. - Increase in alanine and glutamine levels suggests increased protein breakdown, possibly in lung tissue, during COVID-19 infection(↑). Significant differences in amino acid levels between ICU patients and ward patients, with higher levels of glutamine, glutamic acid, histidine, serine, tryptophan, anserine, and taurine in ICU patients. - Positive correlation between amino acid levels and disease severity (*r* = 0.937; *p* < 0.0001).
Anthony T. Le.(2022)	LC/MS-MS	Arginine Metabolism, Tryptophan Metabolism	Aspartic Acid, Sulfocysteine (↑) .Arginine, 3-Methylhistidine, Creatinine, Tryptophan (↓)	Yes	Accuracy (Diagnostic Performance) ↑, Aspartic Acid ↑, Sulfocysteine ↑, Arginine Metabolism (Pathway) ↑, Clinical Correlation (Outcomes) ↑, Specificity (Biomarker Signature) ↑, Arginine ↓, 3-Methylhistidine ↓, Tryptophan ↓.
Lomova NA (2022)	HPLC-MS	Amniotic Fluid: No significant matches with specific metabolic pathways. Umbilical Cord Blood Plasma: Histidine and β-alanine metabolism. Maternal Venous Blood Plasma: D-glutamine and D-glutamate metabolism, arginine biosynthesis, alanine, aspartate, and glutamate metabolism.	Amniotic Fluid: 1-methylhistidine, 3-methylhistidine, Arginine, Cystathionine, Cystine, Glutamine, Histidine, trans-4-hydroxyproline (↓) Maternal Venous Blood Plasma: 1-methylhistidine, Lysine, Cystine, Glutamic acid, Glutamine(↓) Umbilical Cord Blood Plasma: 1-methylhistidine, β-alanine, Cystine, Histidine(↓)	-	Significant differences in amino acid profiles between COVID-19 patients and healthy controls, with potential implications for systemic inflammation and metabolic changes in the mother-fetus system.
Merve Ergin Tuncay (2022)	LC/MS-MS, Spectrophotometric Assay	Proline Metabolism, Collagen Metabolism	Glutamic Acid(↑), Proline, Hydroxyproline (↓)	Yes	Prolidase enzyme activity was significantly lower in patients.
Sedat Özbay et al. (2023)	LC-MS/MS	Urea Cycle, Arginine Metabolism	Arginine, Argininosuccinate, Aspartic Acid, Citrulline, Glutamine, Lysine, Ornithine (↓)	Yes	Arginine and urea cycle amino acids decreased in COVID-19 patients, indicating disturbances in arginine metabolism and immune response
Hu¨seyin Aydın (2023)	LC-MS/MS	Warburg Effect, Glutaminolysis, Pentose Phosphate Pathway, Malate-Aspartate Shuttle	Glutamate, Methionine (↑), Alanine, Arginine, Argininosuccinic acid, Aspartate, Citrulline, Glutamine, Histidine, Lysine, Threonine (↓).	Yes	-
Ina Maltais-Payette (2023)	HPLC-MS/MS	Related circulating amino acid metabolism, Nitrogen Metabolism	Phenylalanine, Methionine, Aspartate, Isoleucine, Leucine, Glutamate, Asparagine, Valine(↑), Proline, Glutamine, Serine, Glycine, Alanine, Histidine, Tryptophan, Cysteine(↓).	Yes	-
Siqi Ming (2023)	Liquid chromatography tandem mass spectrometry (LC-MS/MS)	Arginine biosynthesis, alanine, aspartate, and glutamate metabolism, arachidonic acid metabolism, linoleic acid metabolism	L-citrulline, L-glutamine, L-alanine, L-aspartic acid, L-phenylalanine,	Metabolic profiles of COVID-19 patients were compared to healthy controls.	L-citrulline and L-glutamine levels negatively correlated with disease severity.

#### Branched-chain amino acids and protein catabolism

BCAAs elevation was observed in a study by Baranovicova et al. [30]. Alterations in BCAAs levels (leucine, isoleucine, and valine) may indicate acceleration in protein catabolism and compensatory responses to energy deficits. Conversely Ozturk [25] and Rees [12], demonstrated depleted BCAA concentrations in severe COVID-19 cases, which presents muscle depletion and metabolic exhaustion in critically ill patients.

According to previous investigations BCAA levels may alter across different subgroups of COVID-19 patients. Broader amino acid profile including beta-alanine, tryptophan, cysteine, methionine, phenylalanine, arginine, and tyrosine was measured in study conducted by Zhang et al. [26]. Significant intercorrelation observed among BCAAs, therefore they may have the potential to serve as plausible biomarkers. However, further analysis focusing on specific subgroups, such as patients with myalgia as reported by Ozturk [25], may be needed to refine our understanding of these relationships.

#### Tryptophan metabolism and immune response

Association of immune responses in SARS-CoV-2 infection and altered tryptophan metabolic pathway has been identified in several studies. Elevated levels of tryptophan and its related metabolites (including indole and indole-3-acrylic acid) in COVID-19 was captured by Kaur and co-workers in COVID-19 patients [29], in the other hand decreased levels of tryptophan was reported by Rees [12], Maltais-Payette [20] and Le [24]. Disease severity, immunological activation, and analytical variability may account for these differences. These observations support the involvement of tryptophan metabolism as well as related lipid and amino acid pathways in the inflammatory response to SARS-CoV-2.

#### Glutamine, glutamate, and related amino acids

Glutamine plays a central role in immune cell proliferation and nitrogen metabolism, and its disruption is a consistent finding in COVID-19. Rees [12] reported that L-glutamine, along with trans-4-hydroxy L-proline, L-proline, L-histidine, and L-citrulline, was significantly reduced. Atila [31] found that along with these Amino Acids, taurine and L-threonine has reduced. Baranovicova [30], has also noted that glutamine levels were lower in severe cases. Ali Ozturk [25] and Özbay et al. [22] also confirmed disruptions in glutamine metabolism resulting in its elevation or reduction, respectively. Impaired glutamine availability is considered as a critical factor in COVID-19 pathophysiology based on the prior evidence. Two studies reported a rise in glutamate levels [20, 21].

#### Phenylalanine and the phenylalanine/tyrosine ratio

Elevated levels of phenylalanine among COVID-19 patients were documented in several studies. Atila [31] and Rees [12] demonstrated that L-phenylalanine significantly increased in COVID patients. While elevated phenylalanine-to-tyrosine ratio was also reported by Baranovicova [30]. Additional studiy by Ina Maltais-Payette [20] is consistent with these findings. These alterations in phenylalanine metabolism appear to reflect a systemic inflammatory state and may serve as robust biomarkers for disease severity.

#### Additional amino acid alterations

In addition to key amino acid pathways that were mentioned previously, several other amino acid alterations were identified throughout the examined studies. Decreased taurine concentration was reported in the study conducted by by Atila and collogues [31]. While the study by Ozturk [25], provided evidence for increased taurine levels in specific patient population. Results from both Atila [31] and Tuncay [23],described reduced hydroxyproline and proline levels which is associated with dysregulated collagen metabolism .Amniotic fluid sample and umbilical cord plasma analysis by NA et al., revealed a rise in beta-alanine levels [27] along with decreased methylhistidines in cord blood plasma and maternal samples [27, 28]. Conversely, Ozturk et al. reported elevated levels of these metabolites which suggests disruptions in muscle protein turnover [25].Other amino acids such as, methionine, asparagine, and serine were involved in more complex metabolic pathways as reported by Aydın [21] and Maltais-Payette [20], which supports the metabolic dysregulation linked to SARS-CoV-2 infection.

### Metabolic pathways and systemic implications

The amino acid alterations which were reported in the included studies, were mapped to a specific metabolic pathway. Generally, Atila [31] linked the amino acid alterations to immune response dysregulation, impaired metabolic pathways, and amino acid catabolism. Disruption to arginine metabolic pathway may be connected to endothelial dysfunction, impaired nitric oxide synthesis, and T-cell dysregulation [12]. Tricarboxylic acid (TCA) cycle and protein catabolism disturbances are associated with immune system function [30]. Lipid and tryptophan metabolism along with wider amino acid metabolic pathway were highlighted by Kaur [29]. Amino acid metabolism as well as modulation of protein synthesis, were emphasized by NA [27]. Zili Zhang [26] identified disruptions in amino acid degradation, oxidative phosphorylation, phagosome function, cholesterol metabolism, as well as complement and coagulation cascades. Ozturk [25] pointed to metabolic regulation and immune response as central themes. Le [24] specifically emphasized the roles of arginine and tryptophan metabolism.

NA et al. [28] demonstrated that umbilical cord plasma alterations were associated with histidine and beta-alanine metabolism focusing on maternal-fetal biology, while maternal plasma samples exhibited disruptions in D-glutamine/ D-glutamate metabolism, arginine biosynthesis, and alanine, aspartate, and glutamate metabolism.Tuncay [23] linked diminished proline and hydroxyproline levels to with impaired collagen synthesis [22]. The key role of the urea cycle and distrupted arginine metabolic pathway, alongside with nitrogen metabolism was evidentary supported by Özbay et al. and Maltais-Payette [20, 22]. Compromised arginine biosynthesis, alanine, aspartate, and glutamate metabolism, in addition to in arachidonic and linoleic acid metabolism was mentioned in the study by Ming [19].

### Compartment-specific and longitudinal findings

Multiple studies have reported tissue specific changes in parallel with systemic amino acid dysregulation. Lomova NA [27, 28] demonstrated that amniotic fluid, maternal plasma, and umbilical cord plasma exhibited significant amino acid alterations, which indicates disruptions in fetal metabolic development and protein synthesis. Moreover, the study by Ming et al. [19] documented decreased levels of L-citrulline and L-glutamine in patients with severe COVID-19.

### Methodological influences and diagnostic performance

13 studies utilized LC–MS/MS to assess high-resolution amino acid profiles, with complementary data from NMR spectroscopy. According to Zhang et al. [26] 92 proteins with altered expression and 375 unique proteins were detected in COVID-19 patients. Many of them were significantly linked to metabolic pathways and coagulation cascades. Furthermore, Le et al. [24] introduced metabolic a biomarker panel comprising decreased arginine and tryptophan levels along with increased aspartic acid and sulfocysteine which yielded high specificity in predicting adverse clinical outcomes.

### Risk of bias and study quality

The quality assessment of all 14 included observational studies was carried out using the Newcastle–Ottawa Scale (NOS). Scores ranged from 4 to 9 out of 9 points: five studies were rated as low quality (4–5/9), eight as moderate quality (6–7/9), and one as high quality (8–9/9). Common limitations included inconsistent adjustment for confounders such as age and sex, lack of sample size justification, and potential selection bias in case-control designs. A summary of the quality assessment for each study is detailed in Table 1.

## Discussion

To improve clarity, the discussion is divided into three sections: biomarker potential, therapeutic implications, and comparison with other infections. The immune system is essential for maintaining homeostasis, particularly during infections. Disruption of immune homeostasis contributes to the pathogenesis of severe diseases, including SARS-CoV-2 infection. This systematic review focuses on significant alterations in amino acid profiles among COVID-19 patients, which can maintain the immune function and metabolic pathways. For instance, a reduction in the levels of BCAAs, including leucine, isoleucine, and valine, has been reported across several studies, which may suggest elevated muscle catabolism and immune dysfunction in critically ill patients [27, 28]. Additionally, a decrease in the levels of glutamine, which is an essential source of energy for immune cells, was documented in several cohort studies; this reduction may lead to suppression of lymphocyte proliferation and cytokine production [20]. Furthermore, arginine depletion, which is associated with endothelial dysfunction and dysregulated nitric oxide synthesis, was noted in both adults and pediatric COVID-19 patients. This finding shows that reduced levels of arginine may significantly contribute to disease severity [9]. The present data highlight the critical role of amino acid metabolism, which affects immune response. Moreover, it refers to the potential therapeutic targets to reduce morbidity and mortality in COVID-19 patients.

Dysregulated amino acid metabolism disrupts cellular energy production and redox homeostasis in COVID-19, impairing immune function. Altered levels of BCAAs, glutamine, and arginine contribute to defects in energy production and mitochondrial function. For instance, reduced levels of BCAAs, such as leucine, isoleucine, and valine, indicate increased muscle catabolism to support gluconeogenesis and ATP production during acute illness [27, 28]. Glutamine is a major precursor for glutathione synthesis and a fundamental substrate for oxidative phosphorylation. Decreased levels of this amino acid result in defective antioxidant defense mechanisms and mitochondrial dysfunction in severely ill patients [9]. Furthermore, endothelial dysfunction and reduced nitric oxide availability, exacerbating oxidative stress and inflammation, can occur as a result of disturbances in arginine metabolism, which is identified by decreased arginine levels and elevated arginase activity [16, 22]. Collectively, these findings emphasize the complicated interaction between amino acid metabolism, energy production, and oxidative balance underlying SARS-CoV-2 infection. Understanding these processes may lead to novel therapeutic strategies aimed at restoring metabolic balance and immune homeostasis.

Altered amino acid profiles are reliable biomarkers of severe COVID-19. Changes in arginine, glutamine, and BCAA levels correlate with disease severity, immune dysfunction, and systemic inflammation. For instance, a reduction in arginine levels is frequently associated with endothelial damage and impaired nitric oxide synthesis in both adults and children [9]. Likewise, disrupted immune cell activity occurs as a result of decreased glutamine levels, while higher phenylalanine levels and an increased Phe/Tyr ratio strongly indicate systemic inflammatory conditions [27]. Profiling the specific amino acid metabolism alterations could facilitate early diagnosis of the high-risk patients, which can lead to rapid and targeted clinical intervention [9, 28].

Glutamine administration, for instance, improves the immune response through supporting immune cell metabolism. Similarly, arginine supplementation could recover endothelial function and stimulate NO synthesis, reducing vascular damage. Moreover, restoring BCAAs may be associated with muscle breakdown and metabolic disturbance observed in severe COVID-19 patients [9, 27]. Taurine supplementation has also been hypothesized to improve both metabolic and immune functions in critically ill patients [28]. These targeted nutritional interventions demonstrate the crucial role of personalized treatment approaches in COVID-19 patient management.

Altered amino acid profiles in COVID-19 share similarities with those of other viral infections but also exhibit distinct features. Reduced arginine levels, associated with endothelial dysfunction and impaired nitric oxide synthesis, have also been reported in influenza and hepatitis C [10, 11]. This suggests a shared biological pathway in which viral pathogens manipulate host metabolism to facilitate replication and evade the immune responses. Additionally, due to the particular pathophysiological mechanisms of SARS-CoV-2, specific disruption of BCAAs and their link to muscle catabolism are more frequently observed in severe COVID-19 patients, compared to other viral infections [27, 28]. Higher levels of kynurenine are associated with immune suppression and neurological complications, which are observed in severe COVID-19 cases as a result of upregulation in tryptophan levels via the kynurenine pathway. Similarly, this upregulation was documented in HIV and Ebola virus infections [12, 32]. These similarities highlight the common strategies that viruses employ to modulate the host immunological defenses. However, the distinct metabolic alterations, particularly the imbalances in glutamine and arginine metabolism observed in COVID-19, suggest the unique metabolic demands driven by SARS-CoV-2 infection.

Amino acid profiles in patients with systemic inflammation are mainly consistent with those observed in confirmed COVID-19 infection. For example, a reduction in glutamine concentration, which contributes to impaired immune cell function, has been reported in COVID-19, sepsis, and critical illness [20]. Likewise, the phenylalanine to tyrosine ratio, which is a potential marker of systemic inflammation, is increased in both COVID-19 cases and autoimmune conditions such as rheumatoid arthritis and systemic lupus erythematosus [27].

Furthermore, the elevated levels of BCAAs observed in certain patient populations support the findings in metabolic syndrome and insulin-resistant groups, which suggest a plausible connection between metabolic disruption and disease severity [6]. Based on these overlapping laboratory findings across different clinical conditions, dysregulation of metabolic pathways, including altered amino acid profiles, is not exclusively associated with COVID-19 but is also a common response to systemic stress and inflammation.

Different forms of heterogeneity among included studies, such as differences in study populations, methodologies, and analytical tools, were identified as a primary limitation of the present systematic review. For instance, participant demographics, including age range and sex distribution, vary remarkably across the studies, with some particularly focusing on pregnant women or pediatric patients. The methodologies used to profile amino acids were variable; most studies utilize LC-MS/MS, while others were based on NMR spectroscopy or molecular dynamics simulations [27]. Variabilities in participant characteristics and methodological approaches are followed by discrepancies in the reported amino acid profiles, complicating the synthesis of a clear understanding of metabolic changes in COVID-19 patients.

The other limitation of this review is the inclusion of several studies with fewer than 10 participants, for which the findings are not reliable and cannot be generalized. However, due to the limited number of available studies on this subject, we decided to keep all the studies meeting the inclusion criteria to provide a comprehensive overview of the existing literature. This limitation has been noted, and further investigations with larger, well-designed samples are needed to develop the evidence base.

Moreover, there were no data from patient follow-up to provide insights into dynamic alterations in amino acid profiles during SARS-CoV-2 infection, which is considered another limitation of this review. The time of sample collection was different among the studies; most measured the amino acid levels at a single time point, commonly at the time of hospital admission or during acute illness [9, 20]. Only a few studies attempted to follow up patients, and even these were limited in scope and short duration [27]. Without repeated measurements, it is difficult to clarify whether the observed amino acid disruptions are driven by disease severity or represent the host’s response to systemic inflammation. These limitations have been noted, and further investigations with larger, well-designed samples and long-term follow-up study designs are needed to develop the evidence base of temporal mechanisms of amino acid profile alteration.

Although this review identified significant alterations in amino acid profiles in COVID-19, the underlying biological pathways remain unclear. Future studies should clarify the molecular and cellular mechanisms by which SARS-CoV-2 induces metabolic remodeling. For instance, the way that the virus interferes with host cell machinery and disrupts arginine metabolism, tryptophan catabolism, and BCAA levels could provide insights into the pathophysiology of disease [9, 27]. Advanced techniques, including metabolomics, transcriptomics, and proteomics, alongside with mechanistic studies, could reveal the complex interaction between viral infection and host metabolism.

The observed disruption of amino acid metabolism in COVID-19 patients represents an opportunity for therapeutic intervention. For future investigations, interventional studies should be focused on to assess the potential role of specific amino acid administration in reducing disease severity and improving patient outcomes. In particular, glutamine supplementation could improve immune cell function and reduce systemic inflammation, whereas arginine administration might restore endothelial function and promote NO production [9, 28]. Measuring the levels of these key amino acids could lead to the early identification of high-risk patients and allow early and targeted interventions to prevent severe clinical outcomes. Randomized controlled trials (RCTs) are required to assess the safety, effective dose, and clinical advantages of these therapeutic interventions, particularly in high-risk populations.

Most of the included studies in this review focused on adult populations, which has led to significant gaps in our understanding of amino acid metabolism in pediatric, elderly, and patients with chronic conditions. For instance, due to children’s immature immune system and absence of chronic conditions, different metabolic responses may be demonstrated [9]. In contrast, elderly individuals often suffer from age-related declines in metabolic homeostasis, which could aggravate the amino acid imbalances during infection [6]. Furthermore, distinct metabolic profiles are observed in patients with underlying clinical conditions such as diabetes, cardiovascular disease, or chronic kidney disease that influence disease progression and treatment response [24]. Expanding research to include the wide spectrum of populations will provide a more comprehensive understanding of amino acid metabolism in the context of SARS-CoV-2 infection and inform personalized treatment strategies.

Due to dysregulated amino acid metabolism profile in COVID-19, nutritional interventions could represent a plausible therapeutic role in supportive care for COVID-19 patients. For instance, arginine and glutamine may support immune system function and endothelial health, while BCAA administration could counteract muscle catabolism. Personalized nutritional regimens based on individual amino acid deficiencies may have a favorable prognostic impact on COVID-19 patients and attenuate disease severity.

Nevertheless, excessive amino acid intake particularly in patients with underlying renal or hepatic dysfunction, may have adverse effects; thus, such interventions require careful clinical monitoring.

The observed associations between amino acid alterations and disease severity do not necessarily indicate causality. Further longitudinal and mechanistic studies are required to determine whether these metabolic changes drive immune dysregulation or result from it.

## Conclusion

This systematic review demonstrates that COVID-19 is consistently associated with disturbances in amino acid metabolism, particularly involving arginine, glutamine, tryptophan, phenylalanine, and BCAAs. These metabolic alterations may contribute to disease severity and represent promising biomarkers and therapeutic targets. Future well-designed multicenter studies using standardized metabolomic methodologies are needed to validate these findings and facilitate clinical translation. Future investigations should adopt standardized metabolomic protocols, including harmonized sample collection, analytical platforms, and data-processing pipelines, to improve reproducibility and facilitate comparisons across studies.

## Electronic Supplementary Material

Below is the link to the electronic supplementary material.

Table 3. Quality assessment of included studies using Newcastle–Ottawa Scale Risk of Bias Tool.

## Data Availability

No datasets were generated or analysed during the current study.

## Abbreviations

PRISMA: Preferred Reporting Items for Systematic Reviews and Meta-Analyses
SARS-CoV-2: Severe Acute Respiratory Syndrome Coronavirus 2
COVID-19: Coronavirus Disease 2019
BCAAs: Branched-Chain Amino Acids
NO: Nitric Oxide
LC–MS/MS: Liquid Chromatography–Tandem Mass Spectrometry
NMR: Nuclear Magnetic Resonance
MIS-C: Multisystem Inflammatory Syndrome in Children
TCA: Tricarboxylic Acid
UPLC-MS: Ultra-Performance Liquid Chromatography/Tandem Mass Spectrometry
HPLC-MS: High-Performance Liquid Chromatography-Mass Spectrometry
Phe/Tyr ratio: Phenylalanine-to-tyrosine ratio
RCTs: Randomized Controlled Trials
ATP: Adenosine triphosphate
BMI: Body Mass Index
CRP: C-Reactive Protein
GABR: Global Arginine Bioavailability Ratio
GO: Gene Ontology
ICU: Intensive Care Unit
KEGG: Kyoto Encyclopedia of Genes and Genomes
NOS: Newcastle–Ottawa Scale
PCT: Procalcitonin
