# Supplementary material for: Systematic review of amino acid profiles among COVID-19 patients caused by SARS-CoV-2
Source: J Diabetes Metab Disord. 2026 Jul 16;25(2):201. doi: 10.1007/s40200-026-02012-4 (PMC13375984; doi:10.1007/s40200-026-02012-4)
Supplement: Supplementary file 1 — Table 3. Quality assessment of included studies using Newcastle–Ottawa Scale Risk of Bias Tool. [file 40200_2026_2012_MOESM1_ESM.docx]

**Table 3.** Quality assessment of included studies using Newcastle–Ottawa Scale Risk of Bias Tool.

| **Study (Author, Year)** | **Study Type** | **Assessment Tool** | **Selection** | **Comparability** | **Outcome/Exposure** | **Total Score / Risk** | **Overall Quality** |
| --- | --- | --- | --- | --- | --- | --- | --- |
| Alptug Atila (2021) | Case–control | NOS | 3 | 0 | 2 | 5/9 | Low |
| Chris A. Rees (2021) | Observational | NOS | 3 | 0 | 2 | 5/9 | Low |
| Eva Baranovicova (2021) | Longitudinal | NOS | 3 | 2 | 2 | 7/9 | Moderate |
| Gagandeep Kaur (2021) | Comparative | NOS | 2 | 1 | 2 | 5/9 | Low |
| Lomova NA (2021) | Observational | NOS | 3 | 2 | 2 | 7/9 | Moderate |
| Zili Zhang (2021) | Observational | NOS | 2 | 2 | 2 | 6/9 | Moderate |
| Ali Ozturk (2022) | Case-control | NOS | 3 | 0 | 3 | 6/9 | Moderate |
| Anthony T. Le (2022) | Case-control | NOS | 2 | 2 | 2 | 6/9 | Moderate |
| Lomova NA (2022) | Observational Case-Control | NOS | 3 | 2 | 2 | 7/9 | Moderate |
| Merve Ergin Tuncay (2022) | Case–control | NOS | 3 | 0 | 2 | 5/9 | Low |
| Sedat Özbay (2023) | Cross-sectional | NOS | 3 | 2 | 2 | 7/9 | Moderate |
| Hüseyin Aydın (2023) | Observational | NOS | 3 | 2 | 2 | 7/9 | Moderate |
| Ina Maltais-Payette (2023) | Observational | NOS | 4 | 2 | 3 | 9/9 | High |
| Siqi Ming (2023) | Case-control (cross-sectional) | NOS | 2 | 0 | 2 | 4/9 | Low |
